# Medication Recommendation, Counseling, and Pricing for Nasal Sprays in German Community Pharmacies: A Simulated Patient Investigation

**DOI:** 10.3390/arm93030018

**Published:** 2025-06-13

**Authors:** Bernhard Langer, Christian Kunow, Tim Dethloff, Sarah George

**Affiliations:** Department of Health, Nursing, Management, Neubrandenburg University of Applied Sciences, Brodaer Straße 2, 17033 Neubrandenburg, Germany; christiankunow@googlemail.com (C.K.); tim.dethloff@web.de (T.D.); sarah.geo98@googlemail.com (S.G.)

**Keywords:** self-medication, simulated patient, community pharmacies, medication recommendation, counseling, pricing, nasal sprays, acute rhinosinusitis, common cold, rhinitis medicamentosa

## Abstract

**Highlights:**

**What are the main findings?**

- Slightly more than half of the recommended nasal sprays were free of preservatives.
- The median counseling score was 2.0 out of 8 points, with a significantly higher score observed for a female simulated patient.

**What is the implication of the main finding?**

- Nasal sprays with preservatives were frequently recommended contrary to the medicine guidelines.
- Counseling was poor overall, especially for a male simulated patient.

**Abstract:**

For the self-medication of nasal congestion following a common cold, decongestant nasal sprays can be recommended according to the medicine guidelines. In Germany, these are only available in community pharmacies (CPs) with free pricing. The aim was to analyze the medication recommendation, counseling, and pricing. A covert simulated patient study, internationally recognized as the “gold standard”, was conducted in all CPs of two medium-sized cities in north-eastern Germany. Each CP was visited twice (86 visits) with the identical scenario by one female and one male simulated patient. At the beginning, they asked for a nasal spray and, when asked, stated that they had had nasal congestion for three days. Slightly more than half (54.7%, 47/86) of the recommended nasal sprays were free of preservatives. The median counseling score was 2.0 out of 8 points, with a significantly higher score observed for the female SP (*p* = 0.004). Information on the maximum intake duration of five days recommended in the German pharmacy guideline was not provided during any visits. The prices varied in total from EUR 1.95 to EUR 6.22. Therefore, measures by the legislator and the chambers of pharmacists are necessary to sustainably improve the medication recommendation, the counseling, and the price transparency.

## 1. Introduction

Upper respiratory infections (URIs) have the highest incidence of all causes of disease globally [1]. In 2021, there were 12.8 billion new episodes of URIs [2]. The majority of these mostly self-limiting URIs are caused by viruses [3,4]. These URIs include acute rhinosinusitis [4,5], although the international medicine guidelines do not have a uniform definition. For example, acute rhinosinusitis in viral form is also defined as a common cold by the European position paper [6], in contrast to the international consensus statement [7]. The common cold lasts less than ten days and peaks after three days [6]. Adults have an average of 2 to 4 episodes annually [8,9]. According to the German medicine guideline, which is based on national and international medicine guidelines, a typical symptom may be nasal breathing obstruction caused by nasal congestion [10]. Therefore, topical nasal decongestants [11,12], including decongestant nasal sprays, are recommended [10], also due to the lack of a causal therapy [13].

As the corresponding topical nasal decongestants are available in Germany without prescription as over-the-counter (OTC) medications, but exclusively in community pharmacies (CPs), CPs and the counseling of the pharmacy staff in the context of self-medication have a decisive safety function [14]. In addition to pharmacists, non-pharmacists (e.g., pharmacy technicians) may also provide advice if the pharmacy manager has specified this in advance [15]. In addition, German CPs are legally obliged to ensure “adequate” counseling [15]. For this reason, the German Federal Chamber of Pharmacists (BAK) provides guidance on counseling for numerous indications, including a guideline on self-medication for rhinitis (BAK guideline) [16]. In addition to questions on information gathering (e.g., what symptoms occur), the pharmacy staff must also advise on the recommended medication. In the case of OTC topical nasal decongestants, the focus is on information about duration, as long-term use can lead to rhinitis medicamentosa [17]. In addition to the legal obligation, counseling is relevant for OTC topical nasal decongestants because it is the second-biggest product group in the German pharmacy market [18].

Whether counseling in everyday pharmacy practice meets the requirements of the BAK guideline can be determined using the simulated patient methodology (SPM), which is internationally recognized as the “gold standard” [19,20,21] and is already frequently used in CP practice [22], as a form of covert participatory observation [23]. In this process, a person, who ideally cannot be distinguished from a real customer (simulated patients, SPs), visits a CP and simulates participation in an apparently real consultation situation based on a previously defined scenario to subsequently assess it using previously defined assessment items and, thus, uncover any deficiencies [24]. In contrast to numerous SPM studies in other countries [25,26,27,28,29,30,31,32,33,34,35,36,37,38,39,40,41,42,43,44,45,46], counseling for the common cold has not yet been investigated in Germany. There are only two SPM studies on counseling for nasal sprays, both conducted exclusively in Finnish CPs, with only one being current [47,48]. However, the current SPM studies on counseling for Germany relate to other OTC medications or indications, whereby considerable counseling deficits were found [49,50,51,52,53,54,55].

According to the Joint International Pharmaceutical Federation (FIP)/World Health Organization (WHO) guideline [56], good pharmacy practice should include aspects of the selection and costs of medications in addition to counseling. This raises the question of whether, and if so, which, nasal sprays are recommended and whether they are suitable for the symptom simulated in the scenario. In addition to the selection of nasal sprays with active ingredients (single-drug substances or fixed-dose combinations) in accordance with the medicine guidelines, those with preservatives (e.g., benzalkonium chloride) should be avoided due to possible irritant and harmful effects [10]. The aspect of selection, in addition to the lack of international SPM studies, has so far only been investigated in Germany for other OTC medications or indications [50,52,57], whereby a medication recommendation in accordance with the respective guidelines has not always been shown. On the other hand, the question of costs arises, as the recommendation of comparatively expensive medications (without corresponding additional benefits) represents poor counseling from the customers’ point of view [56,58]. In Germany, OTC medications are generally paid for by the customer without the costs being covered by the health insurance company, and the prices are freely determined [59]. However, price transparency in German CPs is low due to the lack of mandatory price labeling [60] and due to price communication, which usually only takes place shortly before the drug is dispensed [55]. It is, therefore, particularly interesting to see the extent to which price ranges exist that are considered to be the result of a lack of price transparency [61,62]. This applies even more so when price ranges occur in the same city and, thus, in a narrowly defined area with economically identical starting conditions. The aspect of costs, in addition to another lack of international SPM studies, has also only been investigated in Germany for other OTC medications or indications [49,54,57,63], with considerable price ranges being identified.

To close these research gaps, the primary aim of this study was to examine, for nasal sprays,

- which medications are selected (medication recommendation),
- whether questions on information gathering are asked beforehand, and appropriate information is provided on the recommended medications (counseling), and
- the extent to which the costs of the recommended medications differ (pricing).

Secondarily, numerous possible influencing factors on the primary outcomes of the study were to be investigated.

## 2. Methods

### 2.1. Design

The cross-sectional design of the study was based on the SPM. This SPM study was reported according to the STROBE Statement [64] and, building on this, according to the guidelines for health care simulation research specific to SPM [65] and the “Checklist for Reporting research using a Simulated Patient Methodology in Health” (CRiSPHe) [66].

### 2.2. Simulated Patients

In this SPM study, one female and one male master’s student of the Department of Health, Nursing, Management of the Neubrandenburg University of Applied Sciences acted as SPs as part of their three-semester research project, free of charge. At the time of data collection, the female SP was 22 years old, and the male SP was 27 years old. They are both of German ethnicity and had no previous experience as SPs.

### 2.3. Setting and Participation

The SPs visited CPs in the period from 1 October to 30 November 2020 in the second-largest city Schwerin (31 December 2020: 95,609 residents) [67] and in the third-largest city Neubrandenburg (31 December 2020: 63,372 residents) [68] of the north-eastern German federal state of Mecklenburg-Western Pomerania (31 December 2020: approx. 1.61 million residents; 23,294 km^2^ area; low population density of 69.1 residents/km^2^ [69]). All CPs that could be identified in the two towns as of 1 August 2020, on the basis of the pharmacy finder on the “Apotheken-Umschau.de” website [70], were determined in advance. These hits were validated with a corresponding Google search and with a personal visit to all identified CPs one month before the start of the data collection. As a result, a total of 43 CPs (25 CPs in Schwerin and 18 CPs in Neubrandenburg) were included.

### 2.4. Scenario and Assessment

National and international medicine guidelines [6,10] and, in particular, the BAK guideline [16] formed the basis for the scenario (Table 1) and for the assessment items (Table 2). The SPs simulated a product-based scenario by asking about a nasal spray at the beginning of the conversation without having a particular product in mind. The reason for a product-based rather than a symptom-based scenario was that the medication recommendation, counseling, and pricing of nasal sprays should be investigated, and the pharmacy staff should be specifically directed to this. In the case of possible questions for information gathering by the pharmacy staff, the SPs provided the relevant information. The scenario was designed as a self-purchase in that the SPs answered the question “Who is the medication for?” by saying that the medication is for themselves. In addition, it was designed as a “normal” scenario, whereby the limits of self-medication, i.e., a possible recommendation to visit a doctor, were not to be exceeded by the pharmacy staff. For example, when asked the relevant questions, the SPs indicated nasal congestion that had been present for three days based on a common cold [6] without other medical conditions and medications, whereby the presence of a single symptom is in line with the German medicine guideline, which mentions several possible symptoms [10]. The aim was to differentiate it [71,72,73] from other medications (e.g., analgesics, antibiotics (prescription-only in Germany)) and, in particular, from other indications (e.g., allergic rhinitis, COVID-19). Finally, a decongestant nasal spray should always be recommended. This created the prerequisite for checking as comprehensively as possible which nasal spray is selected, whether questions are asked beforehand and corresponding information is provided on the recommended nasal spray and to what extent the costs of the recommended nasal sprays differ.

The assessment comprised 12 items, with the first 5 items assessing whether appropriate questions were asked by the pharmacy staff. Based on these, it was then assessed whether, and if so, which, medication was recommended by the pharmacy staff. For cases where a recommendation was made, it was assessed whether information about dosage, duration, and side effects (items 6, 7, 10) was provided. If duration information was provided (item 7), the exact duration in days (item 8) was recorded and assessed. It was then evaluated whether this duration is consistent with the BAK guideline, valid at the time of data collection, of no longer than five days (item 9). In addition, the trade name (item 11)—to be able to determine the composition (active ingredients, possible preservatives) after the visits on the Internet—and the price (item 12) of the recommended medication were recorded and assessed.

Only objective items were used for the assessment to avoid a subjective assessment by the SPs (e.g., on the friendliness of the pharmacy staff). The categorical items were also completed exclusively using dichotomous scales (closed yes/no questions) so that the SPs had as little room for judgment as possible.

### 2.5. Data Collection

Before the data collection began, the SPs completed comprehensive training. The SPs first familiarized themselves with the theoretical basics of the SPM as well as the scenario and the assessment items. The SPs then carried out four validation visits each so that they could practice using the SPM and confirm the functionality of the scenario and the assessment items. A total of eight validation visits were carried out in CPs outside Schwerin and Neubrandenburg. After the validation visits, a workshop was held to share experiences and to inform each other about the specifics of the scenario and the assessment items. It was found that no changes were needed to the scenario or the assessment items.

Each CP was visited twice to increase the number of visits and, thus, the accuracy of the study results (a total of 86 visits in a total of 43 CPs). For all 43 CPs included, both the first and second runs of visits took place within two weeks, with a two-week break between the two runs to avoid simulating two identical scenarios in the same CPs in quick succession. The visits of the first run (a total of 43 visits, the male SP 22 visits, the female SP 21 visits) were randomly allocated to the two SPs, whereas, for the second run (again, a total of 43 visits: the female SP 22 visits, the male SP 21 visits), the SPs were assigned to the respective visits in the reverse order to the first run. This ensured that each CP was only visited once by each SP to prevent the risk of detection. The visits were conducted on different days of the week and at different times of day. The SPs described their concerns to the pharmacy staff who first approached them. The SPs only provided further information when asked by the pharmacy staff to ensure that the information provided was consistent.

In addition to the assessment items, the SPs also collected numerous variables before, during, and after the visits that may have an influence on the assessment items, in line with the international literature (Table 3).

To avoid unnecessary medication waste, the SPs canceled their purchase by informing the pharmacy staff that they had forgotten their wallet at home, as has already been acted nationally [52] and internationally [35,79]. This purchase abandonment, as it occurred shortly before the payment process, did not influence the previous counseling. Even if the pharmacy staff had found the purchase abandonment (after the counseling had taken place) during the first visit “suspicious”, this purchase abandonment did not influence the counseling of the second visit, as no purchase abandonment situation was apparent during the visit and, therefore, this second counseling situation was also initially “normal”. In addition, a different SP was used for the second visit. At the end of the second visit, the purchase was canceled again, which could then have been perceived as “suspicious” (especially if the pharmacy staff serving the SP was identical). However, this did not affect the counseling or its assessment, since the second counseling situation had already been completed when the purchase was canceled again.

After evaluating the data, each CP received written pharmacy-specific performance feedback including graphically prepared benchmarking, which showed the competitive position of each CP in comparison to the other CPs presented anonymously with regard to the individual assessment items. This gave the CPs the opportunity—if necessary—to initiate appropriate optimization processes with the aim of sustainably increasing counseling.

Since CPs in Germany are classified as system-relevant and must, therefore, remain accessible [80], the COVID-19 pandemic should not have had any influence on the implementation of the data collection.

### 2.6. Data Management and Analysis

The data were entered using the four-eyes principle and analyzed with SPSS version 29 for Windows (IBM, Armonk, NY, USA). All dichotomous question items (items 1 to 5) and three dichotomous information items (items 6, 7, 10) were added up to a “counseling score” (min.: 0 points; max.: 8 points). Items 8 and 9 were not included in the score, as these are a specification of item 7 and not independent counseling items. Items 11 and 12 were not included in the score, as these are not counseling items. The individual items were not weighted, as this would depend on subjective considerations.

As part of the descriptive statistics, frequencies and percentages were determined for the categorical data. The application of the Kolmogorov–Smirnov test and the Shapiro–Wilk test showed that the continuous variables “counseling score” and “price” are not normally distributed. Therefore, the median, interquartile range (IQR), minimum, maximum, and range were calculated. In addition, 95% confidence intervals were reported for categorical data and the median using bootstrapping. The Chi-square test (or, for expected cell frequencies below five, the Fisher [81] or the Fisher–Freeman–Halton exact test [82]) was used to identify possible correlations between the categorical variables of the assessment items (Table 2) and the possible influencing factors (Table 3). Cramer’s V was reported as the effect size measure. For significant results of the Fisher–Freeman–Halton exact test for contingency tables larger than 2 × 2, post-hoc analyses with z-tests for independent proportions using a Benjamini–Hochberg adjustment for multiple comparisons were performed. The Mann–Whitney U-test (effect size Pearson’s r) was used to determine whether the price differed between nasal sprays with and without preservatives and between nasal sprays with single-drug substances and fixed-dose combinations. Using the Mann–Whitney U-test (effect size Pearson’s r) and the Kruskal–Wallis test (analogous to [81], no effect size was reported for more than one degree of freedom), we also analyzed whether the “counseling score” and the price differed with regard to the possible influencing factors. A two-tailed *p*-value of less than 0.05 was considered significant in all statistical analyses.

### 2.7. Ethical Approval

In accordance with the “Guideline for the Use of Mystery Research in Market and Social Research” [83], the data were prepared in such a way that the pharmacy staff of the CPs included in the study could not be identified. To avoid a “Hawthorne effect” [84] and also a possible selection bias [85], the visits were conducted in a concealed manner analogous to international SPM studies [24] and without the possibility of an opt-out. However, to take into account the CPs’ need for information and to ensure informed consent, a letter was sent to all included CPs in March 2020—in line with the recommendation in the international literature [85] and the implementation in numerous SPM studies, e.g., [86,87,88]—providing information about the background and conduct of the study. However, specific information about the planned scenario was not provided in order to avoid jeopardizing the concealed study design. For the same reason, a correspondingly long period (“visits will take place in 2020”) was specified in this letter instead of a specific date, with the visits being carried out at a time unknown to the CPs. The study protocol was approved by the institutional ethics committee of the Neubrandenburg University of Applied Sciences (registration number: HSNB/165/20). Non-disclosure to participants was considered ethically acceptable by the ethics committee, since data were kept anonymous and none of the visits were audiotaped. Recruited students provided their written informed consent to act as SPs. In addition, the authors agreed in writing to keep all experiences and data collected strictly confidential both during the study and after its completion.

## 3. Results

All 86 planned visits were completed, resulting in a visit completion rate of 100%. Table 4 shows the characteristics of the CPs, the pharmacy staff, the SPs, and the visits. The majority of CPs had a quality certificate (63.9%, 55/86). The pharmacy staff were mostly between 30 and 49 years old (61.6%, 53/86) and predominantly female (86.0%, 74/86). In the determined cases, the proportion of non-pharmacists was considerably higher (53.5%, 46/86) than the proportion of pharmacists (24.4%, 21/86). Due to the study design, half of the visits were conducted by a male SP and half by a female SP. The visits took place over an entire day, with the mornings predominating (43.0%, 37/86). In most cases (75.6%, 65/86), there was no queue during the visits.

During the 86 visits, exactly one nasal spray was recommended per visit, i.e., a total of 86 nasal sprays with the following active ingredients: 95.3% (82/86) xylometazoline hydrochloride and 4.7% (4/86) xylometazoline hydrochloride with dexpanthenol. Slightly more than half (54.7%, 47/86) of the nasal sprays were free of preservatives. The prices of the nasal sprays varied overall from EUR 1.95 to EUR 6.22 (∆ 219%; median EUR 3.24 [IQR EUR 2.43–EUR 4.08]), in Schwerin from EUR 1.95 to EUR 6.22 (∆ 219%; median EUR 3.25 [IQR EUR 2.32–EUR 4.08]), and in Neubrandenburg from EUR 2.19 to EUR 4.95 (∆ 126%; median EUR 3.12 [IQR EUR 2.48–EUR 4.08]). There was a significant price difference between nasal sprays with and without preservatives (Mann–Whitney U-test; U = 263.500, *p* < 0.001, r = 0.612 and, thus, a “large” effect size according to Cohen [89]). Nasal sprays without preservatives had a price range from EUR 1.95 to EUR 6.22 (∆ 219%; median EUR 3.95 [IQR EUR 3.25–EUR 4.08]). For nasal sprays with preservatives, the price range was from EUR 2.00 to EUR 4.39 (∆ 120%; median EUR 2.44 [IQR EUR 2.32–EUR 2.91]). There was also a significant price difference between nasal sprays with single-drug substances (exclusively xylometazoline hydrochloride) and fixed-dose combinations (exclusively xylometazoline hydrochloride with dexpanthenol) (Mann–Whitney U-test; U = 23.500, *p* = 0.004, r = 0.311 and, thus, a “medium” effect size according to Cohen [89]). For nasal sprays with single-drug substances, the price range was from EUR 1.95 to EUR 4.39 (∆ 125%; median EUR 3.12 [IQR EUR 2.37–EUR 4.12]). Fixed-dose combinations had a price range from EUR 3.95 to EUR 6.22 (∆ 57%; median EUR 5.59 [IQR EUR 4.20–EUR 6.22]).

**Figure 1:**
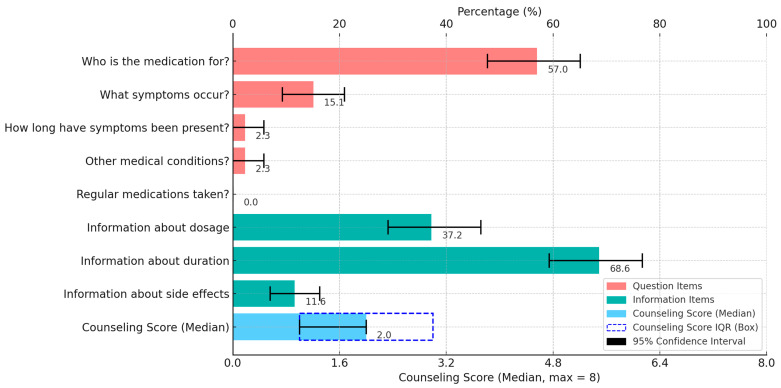
Counseling items and counseling score (*n* = 86).

Overall, the median counseling score was 2.0 out of 8 points (IQR 1.0–3.0). In 16.3% (19/86) of the visits, no counseling took place, i.e., neither were questions asked nor information given by the pharmacy staff. A maximum of six out of eight points was achieved, which was the case in 1.7% (2/86) of the visits. The question “Who is the medication for?” was asked most frequently (57.0%, 49/86). By contrast, the question of which medications are taken regularly was not asked during the visits. The most frequent information was given regarding duration (68.6%, 59/86). Most frequently (86.4%, 51/59), the pharmacy staff referred to a duration of no longer than seven days, in 3.4% (2/59) of visits of no longer than five to seven days, in 5.1% (3/59) of visits of no longer than seven to ten days and in 5.1% (3/59) of visits of no longer than ten days (item 8). The pharmacy staff did not once refer to the duration according to the BAK guideline, valid at the time of data collection, of no longer than five days (item 9). In addition, the least information was provided about side effects (11.6%, 10/86) (Figure 1).

Table 5 shows the associations between the assessment items and the possible influencing factors. It was determined that the female SP was asked significantly more often than the male SP for whom the nasal spray is (Pearson’s chi-square test; χ^2^ = 10.673, *p* = 0.001, V = 0.352, and, thus, a “medium” effect size according to Cohen [89]). The female SP was also significantly more frequently informed about side effects (Pearson’s chi-square test; χ^2^ = 7.242, *p* = 0.007, V = 0.290, and, thus, a “medium” effect size according to Cohen [89]). In addition, there was a significant correlation between the question about the symptoms present and the age of the pharmacy staff (Fisher–Freeman–Halton exact test; *p* = 0.012, V = 0.332, and, thus, a “medium” effect size according to Cohen [89]). A post-hoc analysis showed that the pharmacy staff in the highest age group asked about the symptoms present significantly more often than in the middle age group (Benjamini–Hochberg-adjusted *p* = 0.008). The remaining post-hoc analyses did not lead to significant results. Regarding the “counseling score”, significant differences were found due to the gender of the SPs (Mann–Whitney U-test; U = 602.500, *p* = 0.004, r = 0.306 and, thus, a “medium” effect size according to Cohen [89]). When the female SP was assigned, this led to a median counseling score of 3.0 (IQR 1.0–3.0) with a minimum score of 0 in 14.0% (6/43) and a maximum score of 6 in 2.3% (1/43) of visits. For the male SP, the median counseling score was 1.0 (IQR 0.0–3.0), with a minimum score of 0 in 30.2% (13/43) and a maximum score of 6 in 2.3% (1/43) of visits.

## 4. Discussion

### 4.1. Medication Recommendation

In all visits of the present SPM study, only nasal sprays with active ingredients in accordance with the German [10] and the international [6] medicine guidelines were recommended. However, the BAK guideline—both in the version valid during the data collection and in the current version [16]—does not contain any explicit specifications in this regard. Also, in accordance with the German [10] and the international [6] medicine guideline, not in a single visit—in contrast to SPM studies in other countries with a simulated common cold [29,30,31,32,33,36,37,42,43,44,45,90]—was an antibiotic recommended, although the lack of specifications in this regard in the BAK guideline [16] is understandable due to the prescription requirement for antibiotics in Germany. Nevertheless, the BAK is generally recommended to supplement its pharmacy guideline regarding the recommendation of medications for a common cold in accordance with the medicine guidelines [6,10]. Whether the positive results obtained here would also be seen in a symptom-based scenario, in which SPs would begin the conversation by stating one or more symptoms, should be the subject of future SPM studies.

However, it should be criticized that, contrary to the German medicine guideline [10], nasal sprays with preservatives were recommended in almost half of all visits. However, a different recommendation [91]—in addition to a further lack of specification in the BAK guideline [16]—is opposed by the significantly lower price of nasal sprays with preservatives, from which price-sensitive customers, in particular, can benefit [92], despite the low price elasticity of the pharmaceutical markets [93,94]. For qualitative reasons, however, the BAK is nevertheless urgently recommended to supplement its pharmacy guideline regarding the recommendation of nasal sprays without preservatives in accordance with the German medicine guideline [10]. As a last resort, the German legislator could consider banning nasal sprays with preservatives. This would not be problematic for ensuring supply, as nasal sprays without preservatives are available on the German OTC market, as shown in the other half of the visits [95].

### 4.2. Counseling

Counseling was inadequate overall in the present SPM study, although these results are similar to those of current German SPM studies on the OTC medication loperamide with a mean counseling score from 1.70 (18.9%) to 2.60 (28.9%) out of 9 points [96,97] or for the indications of both acute diarrhea with a mean counseling score from 3.40 (37.8%) to 4.50 (50.0%) out of 9 points [96,97] and non-chronic tension-type headache with a median counseling score of 3.00 out of 9 points [53] and 3.00 out of 12 points [55]. The only two international SPM studies with the question of a nasal spray at the beginning of the conversation also revealed counseling deficits. For example, an older Finnish SPM study determined a mean counseling score from 0.83 (16.6%) to 1.53 (30.6%) out of 5 points at four different assessment times [47]. In comparison, a recent SPM study from Finland showed only slightly better counseling with a mean counseling score of 2.67 (44.5%) out of 6 points [48]. International SPM studies from Slovakia, with the question about zinc medications at the beginning of the conversation with a mean counseling success rate of 39.0% [35], and from Jordan, with the indication of corresponding symptoms also at the beginning of the conversation with a median counseling score of 2.00 out of 15 points [44], also found inadequate counseling in the context of a simulated common cold.

Specifically, duration information was given in almost two-thirds of the visits and, thus, most frequently, as in the current Finnish SPM study, with a similar result of 70.4% [48]. This is not surprising, as this information is extremely important for topical nasal decongestants due to possible rhinitis medicamentosa [17]. However, as stated in the BAK guideline for counseling in the version valid at the time of data collection , no information was provided during any of the visits about a duration of no more than five days. Instead, in most visits, the information given about duration corresponded to the specification of the BAK guideline on drug misuse [17] of no longer than seven days, which was also valid during the data collection, and, in at least three visits, to the specifications of the German [10] and international [6] medicine guidelines of no longer than ten days. This suggests that the pharmacy staff mostly did not comply with the BAK guideline on counseling valid at the time of data collection , but with the BAK guideline on drug misuse [17]. The BAK guideline on counseling has since been updated and now contains the requirement of no longer than seven days [16], but it is, nevertheless, recommended that the BAK be updated again to fit the current requirements of the German [10] and international [6] medicine guidelines. Conversely, as no information on duration was provided in almost a third of visits, both pharmacy staff (through appropriate training by the regional chambers of pharmacists) and customers (through publicly effective campaigns by the German legislator) should be made aware of the enormous importance of this information and compliance with duration.

### 4.3. Influencing Factors

Regarding the secondary objective of this SPM study, it was shown that the gender of the SP influences the primary outcome “counseling score”. The female SP received significantly better counseling than the male SP. Due to the framework conditions (almost identical demographic characteristics of the SPs, standardized answers of the SPs, objective items, completion of the assessment form immediately after leaving the CPs), age, and level of education, as well as different behaviors, different assessment standards or memories of the two SPs can be ruled out as reasons. One reason could be that the (predominantly female) pharmacy staff were interested in more intensive communication with the female SP [98]. It is also possible that the (predominantly female) pharmacy staff thought that the female SP wanted more intensive communication [98]. This could have been enacted in view of the fact that women are generally considered to be more communicative than men, especially when it comes to health issues, particularly in one-on-one communication between women [98,99,100,101]. Nevertheless, the counseling behavior of pharmacy staff is questionable, as the health literacy of men is not only lower than that of women [102,103,104,105], but also generally at a low level [103,104,106]. There is, therefore, a particular need for counseling for this group of people.

Internationally, there are very few comparable SPM studies to date. For example, a study in Qatar found that the two male SPs received significantly better counseling than the two female SPs for the indications of asthma and diabetes. The authors cited the wearing of a burqa by a female SP and the predominantly male pharmacy staff as possible reasons for these surprising results [78]. In contrast, a Spanish SPM study on antibiotic dispensing without prescription showed no significant influence of the different SPs (two women and two men with apparent ages between 30 and 45 years) [107]. However, the outcome “antibiotic dispensing without prescription” is only partially comparable with the outcome “counseling score”. In an Australian SPM study on the indication of infective conjunctivitis, there were no significant gender-specific interaction effects (pharmacy staff and SPs) for the outcomes “noncompliant” and “overtreatment” [108]. Another Australian SPM study on OTC medications for oral emergency contraception showed no differences between male and female SPs regarding the number of counseling points [109]. Due to these different results, there is a need for further research using the SPM and possibly in combination with other methods (for example, interviews).

The fact that older pharmacy staff performed better by asking questions about the symptoms present could be due to their greater knowledge and experience. International SPM studies are contradictory in this regard. While a Malaysian SPM study reported better counseling performance by older pharmacists [110], younger pharmacists performed better in two Australian SPM studies [75,76], so further research is needed.

### 4.4. Pricing

As both national and international SPM studies are lacking regarding the pricing for nasal sprays, it should first be noted that there is a fundamental need for further research. In the present SPM study, the median price in comparison with German SPM studies on other OTC medications is not as high as that for oral emergency contraceptive pills with the active ingredients levonorgestrel (EUR 22.00) and ulipristal acetate (EUR 35.00) [54]. However, this median price is at a similar level to that of OTC medications for acute diarrhea at EUR 2.36 [50] and for non-chronic tension-type headache at EUR 3.46 [57] and EUR 4.95 [55]. The price range in the present SPM study is lower, but still high, compared with German SPM studies on other OTC medications. In other German cities, for example, there are also enormous price ranges for OTC medications for acute diarrhea from EUR 2.36 to EUR 8.49 (Δ 260%) [63] and from EUR 1.99 to EUR 4.53 (Δ 128%) [50], for non-chronic tension-type headache from EUR 0.93 to EUR 9.97 (Δ 972%) [57], and for oral emergency contraceptive pills with the active ingredients levonorgestrel from EUR 10.60 to EUR 32.49 (Δ 207%) and ulipristal acetate from EUR 15.95 to EUR 42.95 (Δ 169%) [54]. This is not just a German phenomenon, as similar patterns were found in international cities. For example, in an Iraqi SPM study in Baghdad on OTC medications for acute diarrhea, the price range was from USD 0.20 to USD 1.38 (∆ 590%) [111]. In a Congolese SPM study in Kinshasa for oral emergency contraceptive pills with the active ingredient levonorgestrel, the price range even extended from USD 0.50 to USD 9.20 (∆ 1.740%) [112].

One reason for price ranges in the present SPM study is that more expensive fixed-dose combinations were recommended to a very small extent. As already shown for OTC medications for non-chronic tension-type headache [57], the median price of fixed-dose combinations is significantly higher than that of nasal sprays with single-drug substances. Another reason is the frequently recommended and significantly more expensive nasal sprays without preservatives. In addition, differences in operating costs between CPs can also occur in the same city (e.g., a cheaper pharmacy location in a suburb and a more expensive pharmacy location in the city center) and be passed on to customers in the form of price ranges. In addition, varying degrees of competition (e.g., many CPs nearby, as observed in the city centers of the cities of Schwerin and Neubrandenburg examined here) can also lead to price ranges [113]. However, differences in the calculation of the respective CPs and, thus, an intention to maximize profits [114] can also contribute to price ranges, especially in the case of market intransparency. However, above a certain price threshold, higher-priced OTC medications can represent a financial burden and, thus, a barrier to access, particularly for low-income earners [115]. Price ranges should, therefore, be reduced by increasing market transparency. The most effective way to do this [116,117] would be to set up a legally binding database with the current prices of CPs, which already exists in Germany for petrol station prices [118]. Such a database could be accessed via an app with corresponding search options such as indications, active ingredients, and preferred shopping locations. It is important to make such an app known to the public through appropriate advertising measures. This is the basis on which such an app works very well, as a German evaluation for petrol station prices [116] has demonstrated.

## 5. Strengths and Limitations

To the authors’ knowledge, this is the first SPM study worldwide, and, thus, also for Germany, to examine medication recommendation, counseling, and pricing for nasal sprays. In contrast to other methods, the use of SPM made it possible to avoid the effect of social desirability. The study could also be implemented as planned. However, it is a cross-sectional study, which means that no causal relationship between the studied variables can be established.

Each CP was visited twice, which should have increased the accuracy of the results. The two visits were conducted by different SPs, who were also of a different gender, which, in addition to minimizing the risk of detection, may also have led to an averaging of the personal characteristics of the SPs and, thus, to the depiction of even more realistic counseling situations. With regard to the gender-specific differences, this study has the advantage over the two studies used for comparison [78,107] that the female and male SPs attended identical CPs, which should have increased the comparability of the results of the male and female SPs.

However, due to limited human resources, the results relate exclusively to two medium-sized cities and, thus, to urban areas in a north-eastern German federal state. However, SPM studies in the same federal state—albeit for other indications—did not show any significant differences between urban and rural areas [51,52]. The results also relate exclusively to a specific scenario for a specific indication, whereby other scenarios [96] or other indications [77] may lead to different results.

Regarding the price ranges determined, important background information on the recommended medications (e.g., package size, originals vs. generics) is missing for the interpretation. This information could not be collected by the SPs on site to avoid the risk of recall bias due to too many items to be collected. As the medications were not purchased, background information could not be obtained retrospectively, e.g., from the package inserts.

In contrast to the most recent German SPM studies [53,55], well over half of the CPs stated that they had a quality certificate. As this item is based on CPs’ self-reports by telephone and not on observations by SPs (not valid for this item) or official figures (e.g., from the regional chambers of pharmacists, which are not publicly available for Mecklenburg-Western Pomerania), a potential for bias cannot be ruled out.

For data protection reasons, (covert) audio recordings for quality assurance during the visits were not used, as otherwise the CPs’ consent would have had to be obtained in advance, which would have given them the option of not participating in the study (opt-out), which, in turn, could have led to selection bias. An alternative option would have been to use a second SP as a second observer. However, as only two SPs were available and each CP was visited twice (by different SPs), there was a lack of necessary human resources.

Finally, immediate performance feedback directly after the respective visit would have been desirable, as the pharmacy staff’s memory of the specific counseling situation would then still have been very present. However, there was a risk that the pharmacy staff would inform CPs in the surrounding area about the visits.

## 6. Conclusions

Contrary to medicine guidelines, nasal sprays with preservatives have often been recommended, which may have irritating and harmful effects. The counseling was poor overall, particularly for the male SP. In addition, there was a wide price range. Therefore, measures of the legislator and the chambers of pharmacists are necessary to sustainably improve medication recommendation, counseling, and price transparency.

## Figures and Tables

**Table 1:** Scenario.

The SP entered the CP and said at the beginning of the conversation: “Hello, a nasal spray, please.” The SP did not have a particular product in mind. When questioned by the pharmacy staff, the following information was provided by the SP:	
**Possible questions on information gathering asked by the pharmacy staff**	**Information given by the SP**
Who is the medication for?	For me
What symptoms occur?	Nasal congestion
How long have the symptoms been present?	For three days
Are there other medical conditions?	No other medical conditions
Which medications are taken regularly?	No medications

Note: If the pharmacy staff asked questions that were not planned in the scenario, the SPs answered “I don’t know”.

**Table 2:** Assessment items.

	YES	NO
1. Pharmacy staff asked: Who is the medication for?	1	0
2. Pharmacy staff asked: What symptoms occur?	1	0
3. Pharmacy staff asked: How long have the symptoms been present?	1	0
4. Pharmacy staff asked: Are there other medical conditions?	1	0
5. Pharmacy staff asked: Which medications are taken regularly?	1	0
6. Pharmacy staff gave information about the dosage	1	0
7. Pharmacy staff gave information about the duration	1	0
8. If 7. Yes: duration (in days)		
9. If 7. Yes: duration according to the BAK guideline [20] of no longer than five days	1	0
10. Pharmacy staff gave information about side effects	1	0
11. Trade name of the recommended medication	Name	
12. Price of the recommended medication	Price (EUR)	

**Table 3:** Possible influencing factors, time, and type of data collection.

Possible Influencing Factors [Literature Sources *]	Time of Data Collection	Type of Data Collection
City of the CPs [74]	Before the visit	Exact measurement by assigning the CPs determined for the respective cities
CP quality certificate [75]	After the visit	Exact measurement using a telephone query by the SPs after completing all the visits
Age of the pharmacy staff [76]	During the visit	Estimate using a visual impression of the SP
Gender of the pharmacy staff [76]	During the visit	Exact measurement using a visual impression of the SP
Professional group of the pharmacy staff [77]	During and after the visit	Exact measurement based on the name tag and, if necessary, using a telephone query by the SP after completing all the visits
Gender of the SPs [78]	Before the visit	Exact measurement based on the gender of the SP
Time of the visit [74]	During the visit	Exact measurement using the SP’s watch
Queue—customers waiting behind the SP [57]	During the visit	Exact measurement using a visual impression of the SP

Note: * The possible influencing factors were taken from the specific literature sources.

**Table 4:** CPs, pharmacy staff, SPs, and visit characteristics.

	Frequency (*n*)	Percentage (%)
All CPs	86	100.0
City of the CPs		
Schwerin	50	58.1
Neubrandenburg	36	41.9
CP quality certificate		
No	31	36.1
Yes	55	63.9
Age of the pharmacy staff		
<30	10	11.6
30–49	53	61.6
≥50	23	26.8
Gender of the pharmacy staff		
Male	12	14.0
Female	74	86.0
Professional group of the pharmacy staff		
Pharmacists	21	24.4
Non-pharmacists	46	53.5
Not able to be determined	19	22.1
Gender of the SPs		
Male	43	50.0
Female	43	50.0
Time of the visit		
8:00 a.m.–12:00 p.m.	37	43.0
12:01 p.m.–4:00 p.m.	26	30.2
4:01 p.m.–8:00 p.m.	23	26.8
Queue—customers waiting behind the SP		
No	65	75.6
Yes	21	24.4

**Table 5:** Associations between assessment items and possible influencing factors (*n* = 86).

	City of the CPs	CP Quality Certificate	Age of the Pharmacy Staff	Gender of the Pharmacy Staff	Professional Group of the Pharmacy Staff	Gender of the SP	Time of the Visit	Queue—Customers Waiting Behind the SP
Medication recommendation	^c^ 0.637 (0.075)	^c^ 0.292 (0.170)	^b^ 0.185 (0.167)	^c^ 1.000 (0.089)	^b^ 1.000 (0.017)	^c^ 0.116 (0.221)	^b^ 0.812 (0.100)	^c^ 0.249 (0.132)
Who is the medication for?	^a^ 0.124 (0.166)	^a^ 0.729 (0.037)	^b^ 0.161 (0.207)	^a^ 0.248 (0.125)	^a^ 0.258 (0.177)	^a^ 0.001 * (0.352)	^a^ 0.433 (0.139)	^a^ 0.986 (0.002)
What symptoms occur?	^a^ 0.342 (0.103)	^c^ 0.540 (0.078)	^b^ 0.012 * (0.332)	^c^ 0.683 (0.076)	^b^ 0.790 (0.069)	^a^ 0.366 (0.097)	^b^ 0.599 (0.106)	^c^ 0.507 (0.089)
How long have the symptoms been present?	^c^ 1.000 (0.025)	^c^ 1.000 (0.041)	^b^ 0.144 (0.255)	^c^ 1.000 (0.062)	^b^ 1.000 (0.144)	^c^ 0.494 (0.154)	^b^ 0.737 (0.111)	^c^ 1.000 (0.088)
Are there other medical conditions?	^c^ 0.172 (0.182)	^c^ 0.999 (0.041)	^b^ 1.000 (0.122)	^c^ 0.261 (0.161)	^b^ 0.717 (0.108)	^c^ 0.494 (0.154)	^b^ 0.504 (0.178)	^c^ 1.000 (0.088)
Information about dosage	^a^ 0.858 (0.019)	^a^ 0.676 (0.045)	^b^ 1.000 (0.034)	^c^ 0.350 (0.107)	^a^ 0.254 (0.179)	^a^ 0.074 (0.192)	^a^ 0.714 (0.089)	^a^ 0.923 (0.010)
Information about duration	^a^ 0.887 (0.015)	^a^ 0.615 (0.054)	^b^ 1.000 (0.019)	^c^ 0.505 (0.089)	^a^ 0.249 (0.180)	^a^ 0.104 (0.175)	^a^ 0.534 (0.121)	^a^ 0.193 (0.140)
Information about side effects	^c^ 0.510 (0.087)	^c^ 1.000 (0.021)	^b^ 0.552 (0.106)	^c^ 1.000 (0.041)	^b^ 0.396 (0.149)	^a^ 0.007 * (0.290)	^b^ 0.104 (0.219)	^c^ 0.701 (0.047)
Counseling score	^d^ 0.403 (0.090)	^d^ 0.739 (0.036)	^e^ 0.409	^d^ 0.500 (0.073)	^e^ 0.228	^d^ 0.004 * (0.306)	^e^ 0.947	^d^ 0.485 (0.075)
Price	^d^ 0.888 (0.015)	^d^ 0.816 (0.025)	^e^ 0.438	^d^ 0.446 (0.082)	^e^ 0.758	^d^ 0.494 (0.074)	^e^ 0.908	^d^ 0.832 (0.023)

Notes: ^a^ Chi-square test *p*-Value (Cramer’s V). ^b^ Fisher–Freeman–Halton exact test *p*-value (Cramer’s V). ^c^ Fisher exact test *p*-Value (Cramer’s V). ^d^ Mann–Whitney U test *p*-value (Pearson’s r). ^e^ Kruskal–Wallis test *p*-Value (analogous to Field [81], the reporting of an effect size for more than one degree of freedom is omitted); * Significant at *p* < 0.05. The question item “Which medications are taken regularly?” and the information item “Duration according to the BAK guideline, valid at the time of data collection, of no longer than five days” were not shown in the table, as no corresponding question was asked or corresponding information provided in any of the visits.

## Data Availability

The datasets are available from the corresponding author upon reasonable request.
